# Machine Learning Reveals a Multipredictor Nomogram for Diagnosing the Alzheimer’s Disease Based on Chemiluminescence Immunoassay for Total Tau in Plasma

**DOI:** 10.3389/fnagi.2022.863673

**Published:** 2022-05-13

**Authors:** Lingyu Zhang, Danhua Wang, Yibei Dai, Xuchu Wang, Ying Cao, Weiwei Liu, Zhihua Tao

**Affiliations:** Department of Laboratory Medicine, The Second Affiliated Hospital of Zhejiang University School of Medicine, Hangzhou, China

**Keywords:** machine learning, nomogram, amnestic mild cognitive impairment, Alzheimer’s disease, chemiluminescence immunoassay, total tau

## Abstract

**Background:**

Predicting amnestic mild cognitive impairment (aMCI) in conversion and Alzheimer’s disease (AD) remains a daunting task. Standard diagnostic procedures for AD population are reliant on neuroimaging features (positron emission tomography, PET), cerebrospinal fluid (CSF) biomarkers (Aβ1-42, T-tau, P-tau), which are expensive or require invasive sampling. The blood-based biomarkers offer the opportunity to provide an alternative approach for easy diagnosis of AD, which would be a less invasive and cost-effective screening tool than currently approved CSF or amyloid β positron emission tomography (PET) biomarkers.

**Methods:**

We developed and validated a sensitive and selective immunoassay for total Tau in plasma. Robust signatures were obtained based on several clinical features selected by multiple machine learning algorithms between the three participant groups. Subsequently, a well-fitted nomogram was constructed and validated, integrating clinical factors and total Tau concentration. The predictive performance was evaluated according to the receiver operating characteristic (ROC) curves and area under the curve (AUC) statistics. Decision curve analysis and calibration curves are used to evaluate the net benefit of nomograms in clinical decision-making.

**Results:**

Under optimum conditions, chemiluminescence analysis (CLIA) displays a desirable dynamic range within Tau concentration from 7.80 to 250 pg/mL with readily achieved higher performances (LOD: 5.16 pg/mL). In the discovery cohort, the discrimination between the three well-defined participant groups according to Tau concentration was in consistent agreement with clinical diagnosis (AD vs. non-MCI: AUC = 0.799; aMCI vs. non-MCI: AUC = 0.691; AD vs. aMCI: AUC = 0.670). Multiple machine learning algorithms identified Age, Gender, EMPG, Tau, ALB, HCY, VB12, and/or Glu as robust signatures. A nomogram integrated total Tau concentration and clinical factors provided better predictive performance (AD vs. non-MCI: AUC = 0.960, AD vs. aMCI: AUC = 0.813 in discovery cohort; AD vs. non-MCI: AUC = 0.938, AD vs. aMCI: AUC = 0.754 in validation cohort).

**Conclusion:**

The developed assay and a satisfactory nomogram model hold promising clinical potential for early diagnosis of aMCI and AD participants.

## Graphical Abstract

Flow diagram of the study design. 
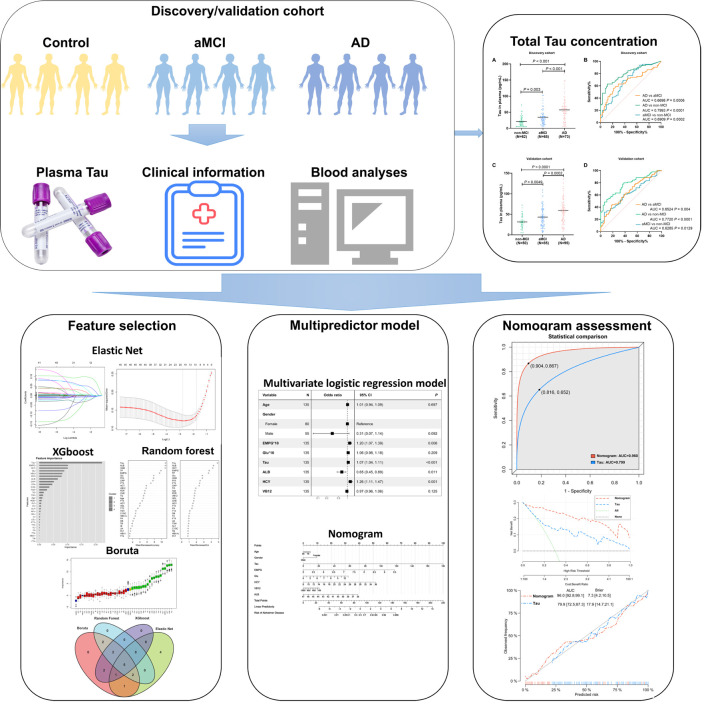

## Introduction

Alzheimer’s disease (AD) is a devastating neurodegenerative disorder in which chronic neuroinflammation results in disease escalation among the elderly. It is characterized by progressive deterioration of cognitive capacity, behavioral and physical disability, and significant and irreversible brain damage (Nelson et al., 2012; Jagust, 2018). The mechanism leading to the initiation and propagation of this neurodegenerative disease remains a topic of intense debate. The neuropathologic hallmarks of AD are deposition of neuritic amyloid plaques (amyloid-β peptide) and intraneuronal accumulation of neurofibrillary tangles (NFTs), followed by toxic protein aggregation and cell loss in defined brain regions (Grothe and Teipel, 2016; Fitzpatrick et al., 2017; Josephs et al., 2017). MCI, particularly the amnestic subtype (aMCI), is usually considered an intermediate stage between normal aging and a diagnosis of clinically probable AD (Whitwell et al., 2007). Amnestic MCI (aMCI) is a subtype in which subjects exhibit distinctive memory impairments, with or without impairment of multiple cognitive domains, but do not meet the criteria for dementia (Huang et al., 2020). Early diagnosis and treatment development of AD would greatly benefit from identifying biomarkers at the prodromal stage, which may modify disease progression (Mueller et al., 2005; Perani et al., 2016). Nowadays, biological measures remain the gold standard for diagnosing AD and assessing MCI conversion. The increasing availability of techniques [i.e., mini-mental state examination (MMSE), cerebrospinal fluid (CSF) biomarker analysis, positron emission tomography (PET) with β -amyloid or tau tracers, brain magnetic resonance imaging (MRI)] able to detect ***in vivo*** AD pathological hallmarks (Santangelo et al., 2020**;** Arevalo-Rodriguez et al., 2021). The clinical diagnosis of AD currently relies on neurophysiological examination and neuroimaging, such as mini-mental state examination (MMSE), activities of daily living (ADL), Montreal Cognitive Assessment test (MoCA), and physiological examination of cerebrospinal fluid (CSF β 1-42, T-tau, P-tau), mainly leading to complementary explorations, such as single-photon emission computed tomography (SPECT) or positron emission tomography (PET; Mattsson et al., 2015, 2018**;** Roalf et al., 2017**;** Li et al., 2018**;** Meyer et al., 2020). Determining amyloid β, total Tau, and phosphorylated Tau-181 in CSF samples has emerged as a powerful tool for AD discrimination due to its stability, sensitivity, and specificity (Mielke et al., 2018**;** Song et al., 2018**;** Janelidze et al., 2020). Some researchers pointed out that mild cognitive impairment in progression and Alzheimer’s disease dementia can be predicted by clinical features and a combination of CSF biomarkers may increase the predictive power (Prestia et al., 2015**;** Tan et al., 2018**;** Spasov et al., 2019**;** Santangelo et al., 2020). However, CSF samples are obtained through a lumbar puncture (LP) or lumbar drainage (LD), which is an invasive procedure with multiple contraindications and treatment-related sequelae, and repeating operations has proven difficult. Besides, PET/SPECT testing is a time-consuming and costly imaging procedure and is unavailable for most situations (Duits et al., 2016). Blood measurements offer advantages over CSF for AD biomarker screening, as blood collection is easier and less invasive. As a result, biomarkers screened from other biological fluid samples have become increasingly fulfilling the needs for clinical diagnostics. Increasingly accessible laboratory techniques applied for the detection of Aβ 40/42 oligomers, total Tau and phosphorylated Tau (P-Tau181/217) have recently been quickly developed, such as multiplex immunoassay, enzyme-linked immunosorbent assay (ELISA), electrochemical detection method, surface-enhanced Raman spectroscopy and localized surface plasmon resonance (LSPR), etc. (Chou et al., 2008; Liu et al., 2015; Xia et al., 2016; Kim et al., 2018, 2019; Yin et al., 2019).

This study aimed to determine the diagnostic accuracy of total Tau in plasma for AD, both for the clinical diagnosis of AD and predicting the probability of suffering from Alzheimer’s disease. This report has developed and internally verified a multipredictor nomogram that combines total plasma Tau concentration and other robust clinical signatures to diagnose AD and aMCI. Relative to individual clinical variables, adding biomarkers substantially improved the diagnostic efficiency through multiple feature selection algorithms (i.e., AD vs. non-MCI, an improvement in predictive accuracy from 79.9 to 96.0%). Before these encouraging findings can be implemented into routine clinical practice, they need to be validated in an independent external cohort, representing this study’s purpose.

## Materials and Methods

### Chemicals and Materials

A full-length recombinant human Tau protein (Tau441, ab84700) ladder was purchased from Abcam (Cambridge, United Kingdom). A pair of specific anti-Tau oligomer antibodies (db7399 and db8769) were provided by DiagBio Co., Ltd. (Hangzhou, China) with a purity of >99%. All polyclonal antibodies (complete IgG) used in our experiments are commercially available. Recombinant human T-tau was reconstituted by 1 **μ** M in 10 mM phosphate-buffered solution (PBS with 0.002% PMSF, 0.03% DTT, and 25% Glycerol, pH 7.4) and stored at −80°C. Acridine-NHS ester (NSP-SA-NHS) and N-hydroxysuccinimide Biotin (NHSB) were purchased from Shchemsky (Shanghai, China). Streptavidin-coupled superparamagnetic nano-magnetic iron particles were acquired from Roche. A 5% bovine serum albumin (BSA; Sigma, Shanghai, China) dissolved in PBS (with 0.05% Tween 20) was immobilized for blocking non-specific binding. An 0.05% PBST solution (PBS with Tween 20; pH 7.4) was utilized as a washing buffer. Deionized water (18 M**Ω** cm, Milli-Q gradient system, Millipore, Darmstadt, Germany) was used throughout the experiments.

### Preparation of Biotin-Bioconjugated Capture Antibody

N-hydroxysuccinimide biotin was prepared by dissolving in dimethyl sulfoxide (DMSO) to 1 mg/mL. Labeled samples were diluted into PBS (pH 7.4) to an appropriate concentration (∼0.5 mg/mL). Capture antibodies (db7399, Rabbit polyclonal) were biotinylated with sulfo-N-hydroxysuccinimide (NHS)-biotin for 30 min at 37°C (molar ratio 1:20) following the manufacturer’s instructions. After the reaction, free NHS-biotin was separated on a PD-10 desalting column (GE Healthcare, Darmstadt, Germany). The labeled antibodies were stored at −20°C after adding appropriate storage solution, and their concentrations were estimated using a Pierce BCA protein assay kit (Life Technologies, Darmstadt, Germany).

### Preparation of AE-Conjugated Detection Antibody

As described in the manufacturer’s instructions, acridine-NHS ester (NSP-SA-NHS, AE) was solubilized in N, N-dimethylformamide (DMF) to prepare a 5 mM AE-NHS solution. The concentration of unconjugated antibody (db8769, Mouse polyclonal) was first adjusted to 1.0 mg/mL using 1 M aqueous sodium bicarbonate (pH = 8.3), and 5 μL DMF solution with NSP-SA-NHS dye was added. These reagents were allowed to react at 30°C for ∼30 min. After the reaction, a G25 size-exclusion gel chromatography column was employed to purify labeled antibodies from the free dye. The column was equilibrated with PBS buffer before adding the reaction mixture to the G25 column. The column was then connected to a fraction collector and eluted with PBS buffer, 500 μL each time. The eluent was collected by continuously monitoring OD value at 280 nm, and 0.1% BSA and 0.05% NaN_3_ (w/v) were added. This labeled antibody was used at a final concentration of 1 μg/mL and stored at −20°C. Unless otherwise stated, the procedures for storage of labeled antibodies are the same.

### Reaction Procedures of the CLIA Assay

**FIGURE 1:**
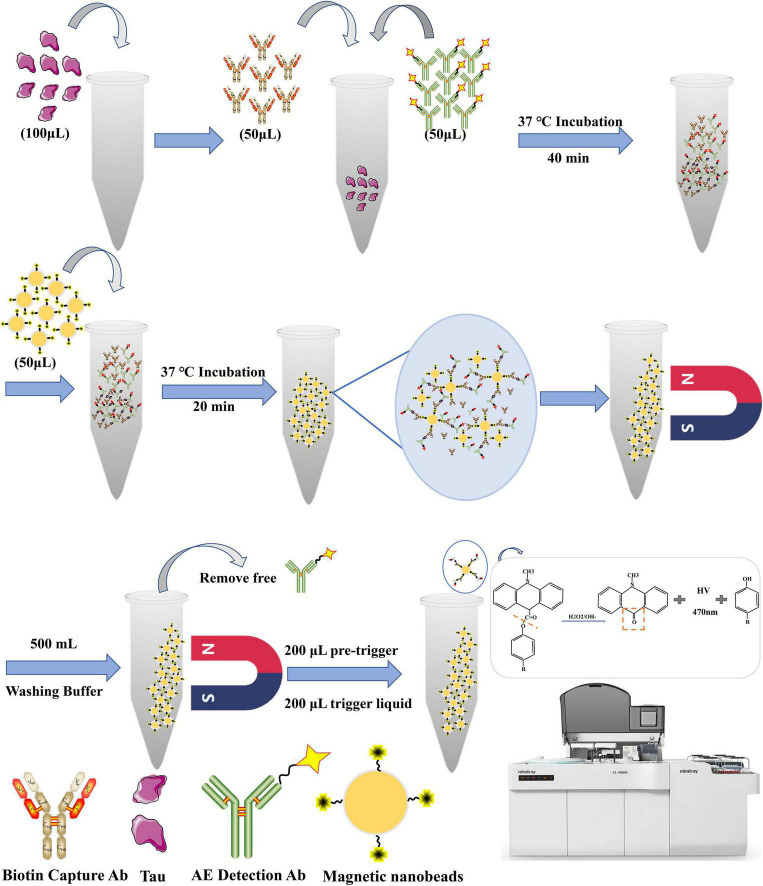
Schematic diagram of CLIA-based technology for total Tau quantification in plasma.

The schematic diagram of the proposed method is illustrated in Figure 1. The assay process involves two uninterrupted incubation steps of 60-min duration. According to experimental design, we set 10 different standard sample concentrations (1000, 500, 250, 125, 62.50, 31.25, 15.63, 7.81, 3.9, and 0 pg/mL). To improve the sensitivity of the detection system, the samples were treated with guanidine hydrochloride (Gua-HCl), revealing the barrier from other proteins occupying the epitope of Tau protein in plasma. During the first incubation, 100 μL of sample is incubated with two polyclonal antibodies specific to Biotin-Ab (50 μL) and AE-Ab (50 μL), respectively, resulting in a sandwich complex formation specific for total Tau. In the following incubation, after adding streptavidin-coated superparamagnetic iron oxide nanoparticles, the sandwich complex becomes bound to the solid phase. Using a magnetic field, the solid and liquid phases are separated. Following that, a thorough rinse with 0.1 M PBS buffer containing 0.05% Tween 20 (pH 7.4) was performed to remove unbound non-reacted antibodies and other biomolecules. In a chemical detection system triggered by NSP-SA-NHS, NaOH (trigger solution) is added as a mediator to transfer the electron between NSP-SA-NHS and hydrogen peroxide (H_2_O_2_) (pre-trigger solution). In an alkaline solution, when hydrogen peroxide ions attack the molecule of acridine ester, the substitution on the acridine ring forms unstable dioxane, which is then decomposed into CO_2_ and electronically excited N-methylacridone (photon emitter) (Fu et al., 2018; Pham et al., 2020; Zhao et al., 2021). A photon is immediately released when the excited N-methylacridone returns to the ground state. During this period, a photomultiplier can monitor and quantify the reduced current [relative light units (RLU)].

### Optimization of CLIA Conditions

Generally speaking, the optimal detection conditions are critical to improving the sensitivity of the assay. Several physicochemical factors that influenced CLIA performance were carefully optimized in this work. Non-specific adsorption is bound to increase background noise and impair the sensitivity of the analysis. Therefore, the appropriate detection antibody concentration is significant. As for capture antibodies, an adequate combination of antigen and antibody is the key to accurate quantification, while excessive capture antibodies can compete with the sandwich complex for streptavidin epitope. Serial dilutions of Biotin-Ab (12.8, 9.6, 6.4, 3.2, 1.6, 0.8, 0.4, 0.2, 0.1, and 0.05 μg/mL) and AE-Ab (dilution ratio: 1:1, 1:2, 1:4, and 1:8) were allowed to react with a series of standard Tau samples and blank samples (PBST with 0.5% BSA) as described earlier. Under pre-designed conditions, we used GraphPad Prism 9 to draw a fitting curve that varies with antibody concentration. We determined the optimal concentration of capture antibody and detection antibody concentration through comprehensive consideration of sample RLU and background noise.

### Assessment of Analytical Performance

*Linearity*: According to experimental requirements, we designed a series of standard Tau sample concentrations (1000, 500, 250, 125, 62.50, 31.25, 15.63, 7.81, 3.9, and 0 pg/mL), and plotted the RLU value that varies with Tau concentration. Deviations from linearity were calculated using linear, quadratic, or cubic regression models. If no higher-order regression was significant, then linearity was accepted (*R*^2^ > 0.99). *Limits and functional sensitivity of the assay*: As recommended by Clinical Laboratory Standards Institute (CLSI) guideline EP17-A2 (CLSI, 2012), multiple tests were performed to determine the low limits of detection (LOD) and limit of quantification (LOQ). Over 2 days, ten blank samples conducted 60 measurements, with each sample repeated three times per day. LOD and LOQ calculations were employed either parametrically or non-parametrically as appropriate (Zhao et al., 2021). *Precision*: Precision testing was also performed following the CLSI EP05-A3 guideline (CLSI, 2014). The standard protocol incarnated a nestification of variance components design with 15 testing days, 3 runs per day, and 2 replicates per measurement (a 15 × 3 × 2 design) for each level (Total number of replicates: 90 per node, Level 1: 10 pg/mL, Level 2: 50 pg/mL, Level 3: 100 pg/mL).

### Study Designs and Participants

A total of 318 patients treated at the Department of Neurology in The Second Affiliated Hospital of Zhejiang University School of Medicine between May 2020 and April 2021 (discovery cohort: 138, 2020.05–2021.01; validation cohort: 180, 2021.01–2021.08) were reviewed and included in this study if the following conditions were met: (i) received high-resolution morphological 18F-FDG PET or MRI to rule out other brain inflammation; (ii) a neurological examination, review of medical history, and administration of Logical Memory Scale-II (LM II) and MMSE; (iii) modified Hachinski ischemia score ≤4; (iv) all patients receive cranial CT or MRI to rule out trauma or inflammation caused by other reasons; (v) no history of cerebrovascular accident such as cerebral hemorrhage or cerebral infarction; (vi) no nervous system tumors or other systemic malignancies; (vii) no history of drug abuse or carbon monoxide poisoning. Clinically, the diagnosis criteria of AD and aMCI followed the recommendations of the NIA-AA Association and the Chinese Classification and Diagnostic Criteria for Mental Disorders (CCMD-3). We recruited age-matched (55∼85 years old) individuals with no cognitive impairment at the physical examination center as healthy controls (discovery cohort: 62; validation cohort: 50). Before enrollment, all participants and/or their parents or legal guardians have been fully informed and completed a written consent form. After fasting for at least 8 h, whole blood samples were collected in tubes containing EDTA-K2 and immediately centrifuged at 3000 *g* for 10 min to obtain cell-free plasma (Rózga et al., 2019; Liu et al., 2020).

### Statistical Analysis

Statistical analyses were applied using SPSS v.26.0 and RStudio v.4.0.1. The data operations from discovery and validation cohorts were mutually independent. Chi-square tests were performed for categorical variables (e.g., gender) and differences in non-normally distributed numerical data using Wilcoxon rank-sum test between subgroups. To screen robust features, Elastic Net [a combination of Ridge and least absolute shrinkage and selection operator (LASSO) method], Random Forest, Boruta, and extreme gradient boosting (XGBoost) analyses were performed to select the most important subgroup-relevant features by calculating the importance score for each variable (Engebretsen and Bohlin, 2019; Johnson et al., 2020; Yperman et al., 2020; Colen et al., 2021; Rahman et al., 2021). With the remaining variables, the predictors were incorporated into a multivariable logistic regression model while adjusting for potential confounders including Age and Gender, considering the effect of participant structure on statistical results. Based on regression analysis results, nomograms are established and estimated using bootstrap sampling with 1000 repetitions and subsequently performed with 10 fold cross-validation. The nomogram and calibration curves were generated with “rms” package of R software^1^, and Hosmer–Lemeshow test was performed to assess goodness-of-fit. Overall goodness-of-fit was assessed with the C-index, Brier score, and calibration using plots of observed versus predicted risk. Decision curve analysis (DCA) is employed to evaluate the net benefit index of the new model. The primary means of assessing the discriminatory performance of the nomogram model or biomarkers was the area under ROC curves (AUC; Iasonos et al., 2008; Shariat et al., 2008). The net reclassification index (NRI) and integrated discrimination improvement (IDI) were deployed to compare the probability differences and quantitative differences between the two models (Kundu et al., 2011; Martens et al., 2019). By comparing NRI and IDI, we determined the better nomogram. The whole process was performed in R 3.3.2^2^. The *P*-values for all hypothesis tests were two-tailed, at a statistical significance level of *P* < 0.05, unless stated otherwise.

## Results

### Optimization of CLIA Conditions

**FIGURE 2:**
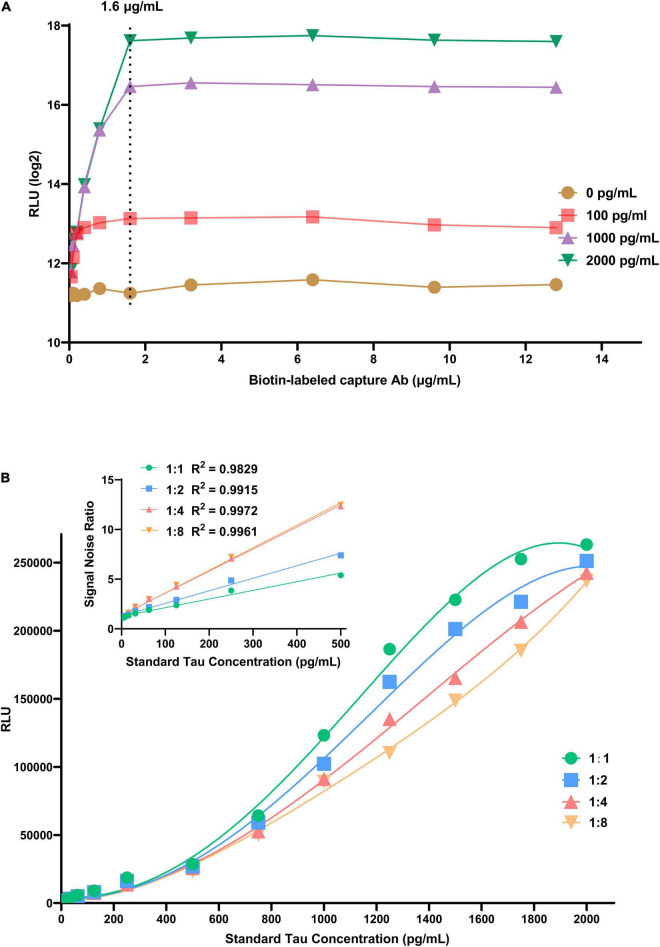
The effects of experimental conditions on CLIA performance. **(A)** The effect of biotin-labeled capture antibody. **(B)** The effect of dilution times of AE-labeled detection antibody.

The concentration of immunosorbent (capture antibody) is a key factor affecting the analysis performance; it can bind all the target antigens in the sample to be tested. Streptavidin-coupled magnetic nanobeads should provide enough epitopes to accommodate the whole sandwich complex. In this experiment, we set up a series of different concentrations of Tau samples (0, 100, 1000, and 2000 pg/mL) and capture antibodies (0.05, 0.10, 0.20, 0.40, 0.80, 1.60, 3.20, 6.40, 9.60, and 12.80 μg/mL). As revealed in Figure 2A, a standard curve was plotted based on chemiluminescence intensity of different Tau concentrations to assess associations between luminescence intensity and capture antibody concentration. The luminescence intensity increases with capture antibody concentration and reaches equilibrium at 1.6 μg/mL. As the concentration continues to increase, the luminescence intensity tends to decrease, which may be caused by excess capture antibodies occupying the streptavidin epitope. Besides, we performed a dilution series of the detection antibody (dilution ratio: 1:1, 1:2, 1:4, and 1:8) to determine how few antibodies are required for detection above background. In all cases, relative luminescence intensity was correlated with the level of detection antibody, implying that the higher concentration, the higher the luminescence intensity. Considering signal-to-noise ratio (SNR) and detectable linear range of the target antigen, we chose 1: 8 as the optimal dilution ratio of the detection antibody (Figure 2B).

### Total Tau Detection Using CLIA

**FIGURE 3:**
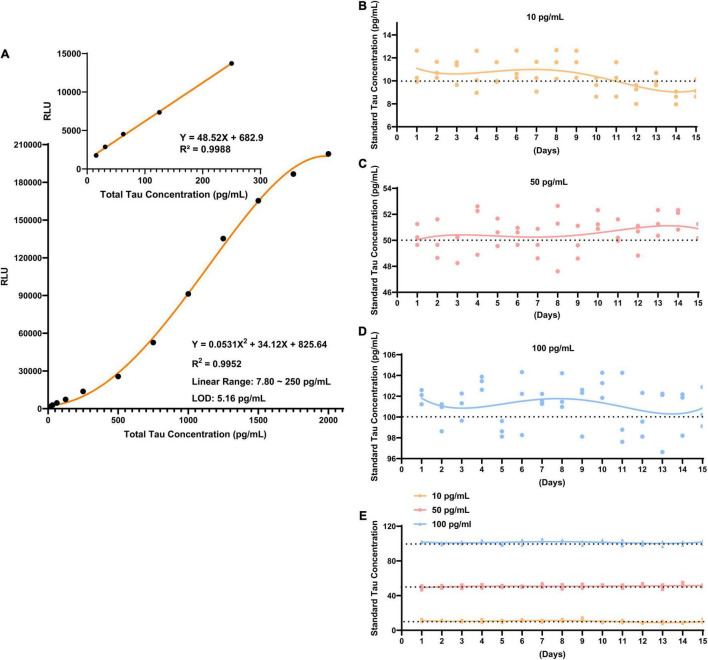
The performance of CLIA for total Tau. **(A)** A quantification of total Tau using developed CLIA. The inset shows a dynamic linear range of Tau concentrations from 7.80 to 250 pg/mL. The repeatability was determined at three standard Tau concentrations: **(B)** 10 pg/mL; **(C)** 50 pg/mL; **(D)** 100 pg/mL. **(E)** Overall landscape of the three concentrations. The error bars expose the standard deviation.

Under optimized conditions, a direct standard CLIA curve was established. The net luminescence intensity (RLU minus background signal noise) varies continuously against various concentrations of Tau standard solution (3.90–2000 pg/mL). The calibration curve of developed CLIA covered a satisfactory linear range from 7.80 to 250 pg/mL with a reliable correlation coefficient (*R*^2^ = 0.9988). In this calibration curve, the limit of detection (LOD) is 5.16 pg/mL (Figure 3A); it is defined as the lowest dilution ratio at which more than 95% of contrived replicates are detected, yielding a signal three times higher than the standard deviation of blank samples. The applicability of the developed methodology to routine application depends on its high reproducibility. Following experimental design, repeatability was investigated by three tests at every Tau concentration in 15 days. The reproducibility of method was assessed by relative standard deviations (RSDs). The RSDs of intra-day precision and inter-day precision for each level of Tau was ≤10%. Supplementary Table 1 displays that average recovery yields of Tau at three levels were all in the range of 85–111% (Level 1: 85–111%; Level 2: 94–104%; Level 3: 98–103%), and their RSD was all ≤10% (Figures 3B–E). Detailed results are reported in Supplementary Table 1. These results emphasized that CLIA exhibited fairly repeatability and reliability compared to other detection technologies for total Tau (Supplementary Table 2) (Kang et al., 2012; Demeritte et al., 2015; Bayart et al., 2019). We deeply realize that although the LOD and linear range of the CLIA method is not as good as other detection techniques, there are certain advantages in the time-consuming and simplified operation process.

### Plasma Total Tau Distribution in Alzheimer’s Disease/Amnestic Mild Cognitive Impairment Patients

**FIGURE 4:**
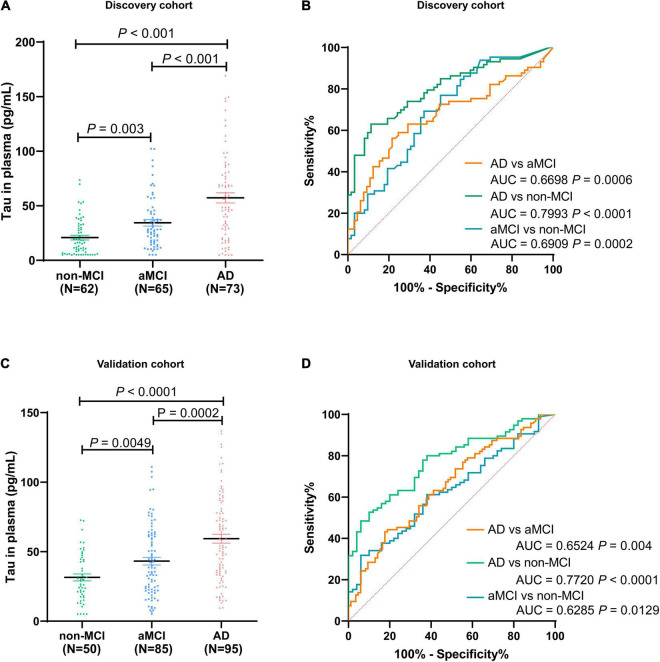
Distribution of total Tau in plasma among non-MCI, aMCI, and AD populations in discovery cohort **(A)** and validation cohort **(C)**. The ROC analyses of total Tau in AD patients, aMCI patients, and non-MCI participants in the discovery cohort **(B)** and validation cohort **(D)**. In the discovery cohort, *n* = 73 (AD), 65 (aMCI), and 62 (non-MCI). In the validation cohort, *n* = 95 (AD), 85 (aMCI), and 50 (non-MCI).

We assessed the level of total Tau in plasma among 200 participants in discovery cohort (non-MCI = 62, aMCI = 65, AD = 73). The Tau contents in plasma of healthy controls (non-MCI) were in the range of 16.55–25.11 pg/mL (95% confidence interval). In contrast, Tau content in plasma of aMCI patients varied from 28.51 to 40.28 pg/mL, and AD patients varied from 47.87 to 66.59 pg/mL (Figure 4A). Participants in AD group had significantly higher total Tau levels than those in aMCI and non-MCI groups, and the increased Tau levels were ordered by clinical group AD > aMCI > non-MCI. To test the ability of Tau as a biomarker to discriminate different participants, we evaluated ROC curves, which assessed the diagnostic performance of a classifier by varying its discrimination threshold. In detail, all these results indicate that total Tau in plasma has a higher ability to distinguish AD patients from non-MCI controls (AUC = 0.7993, *P* < 0.0001), while between aMCI and non-MCI controls, or between AD and aMCI, the performance is moderate (aMCI vs. non-MCI: 0.6909, *P* = 0.0002; AD vs. aMCI: 0.6698, *P* = 0.0006) (Figure 4B). These data indicate that total Tau in plasma performs as promising candidates for AD and aMCI diagnosis. These results were also observed in an independent validation cohort composed of 50 non-MCI, 85 aMCI, and 95 AD participants (Figures 4C,D). Furthermore, several limitations remain in translation to clinical application, its performance is not yet satisfactory for clinical applications.

### Construction and Validation of the Multipredictor Nomogram

**FIGURE 5:**
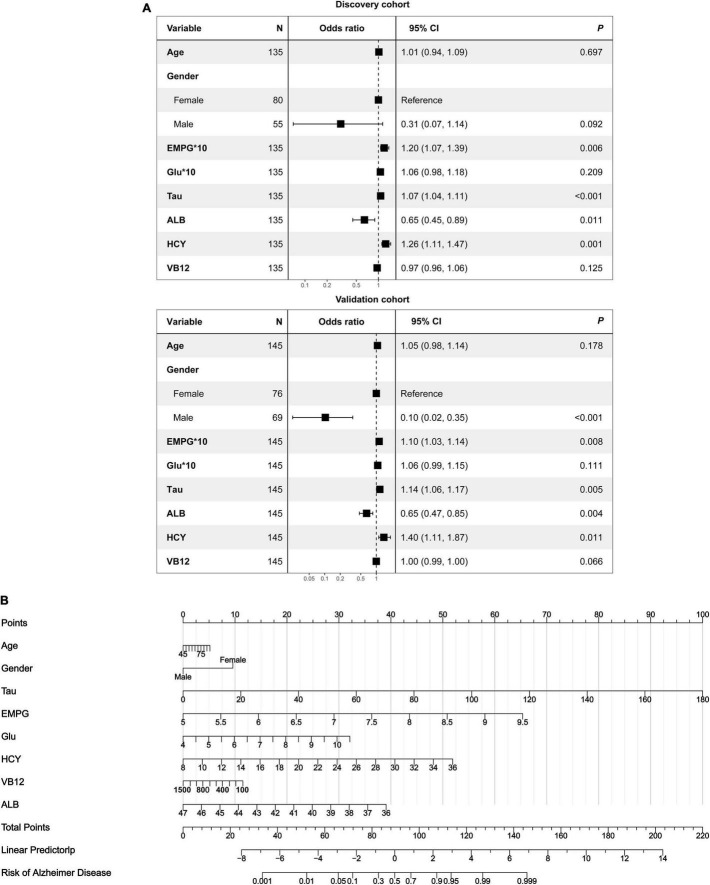
Development of a multipredictor nomogram for AD diagnosis. (A) The forest plot of OR of selected features in discovery and validation cohorts. (B) Predictive nomogram integrates multiple selected features.

**FIGURE 6:**
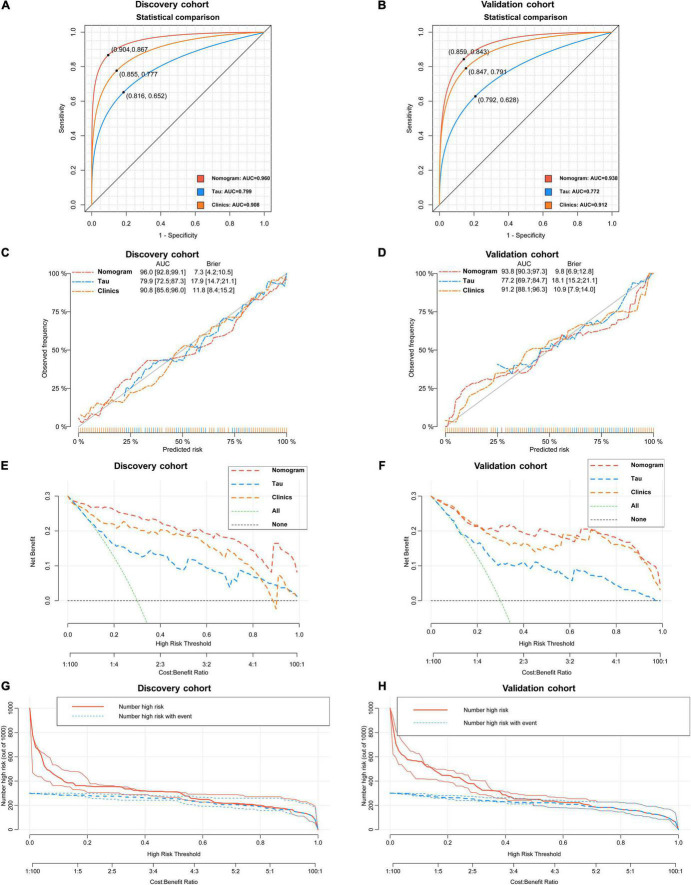
Statistical performance and clinical application of the nomogram. (A) Novel nomogram outperforms total Tau in plasma and Clinical model for discriminating AD patients and non-MCI participants in discovery cohort [(A) 0.960 vs. 0.799 and 0.908, *P* < 0.05] and validation cohort [(B) 0.938 vs. 0.772 and 0.912, *P* < 0.05]. Cross-validated calibration curves of the three models in discovery cohort (C) and validation cohort (D). Decision curve analysis demonstrating the net benefit associated with using the novel nomogram in discovery cohort (E) and validation cohort compared to total and Clinical model (F). Clinical impact curves of the nomogram model in discovery cohort (G) and validation cohort (H). The red curve (number of high-risk participants) indicates the number of people who are identified as AD (high risk) by the nomogram at each threshold probability; the blue curve (number of high-risk with event) is the number of true diagnoses at each threshold probability.

**FIGURE 7:**
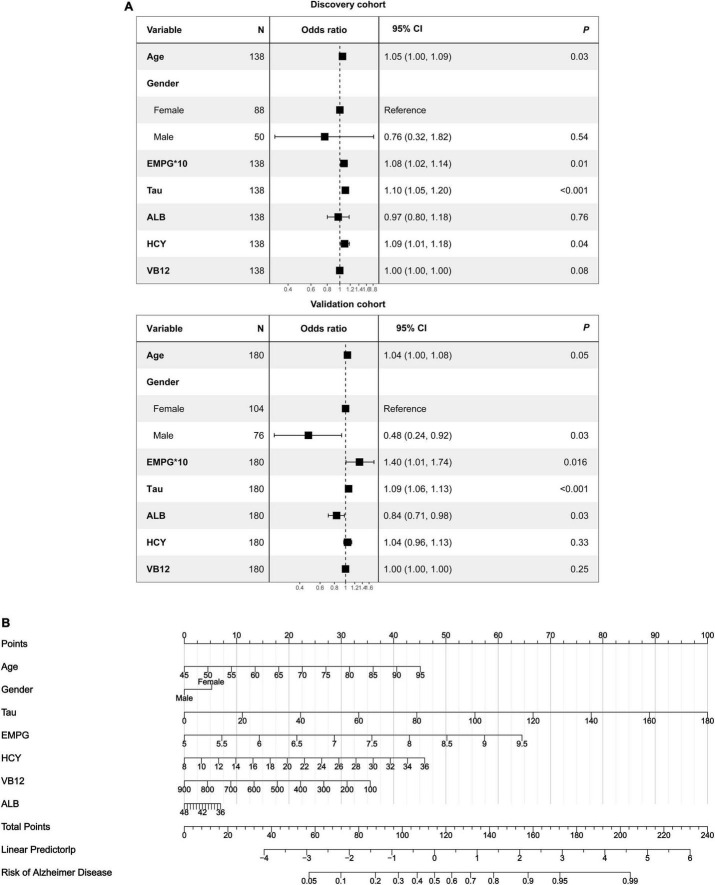
The nomogram for differentiating aMCI and AD. (A) Forest plot of the predictive value of the selected features for multivariable analyses. (B) A predictive nomogram based on clinicopathological features and total Tau.

**FIGURE 8:**
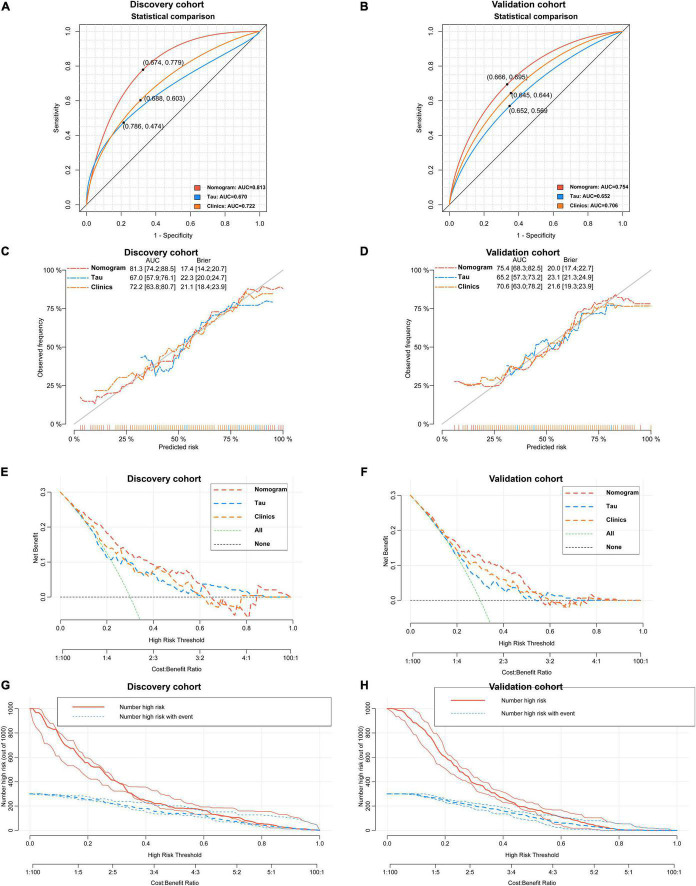
Verification of the predictive performance and clinical applicability of the nomogram for differentiating aMCI and AD. A nomogram outperforms total Tau and Clinical model for predicting the probability of AD in discovery cohort [(A) 0.813 vs. 0.670 and 0.722, *P* < 0.05] and validation cohort [(B) 0.754 vs. 0.652 and 0.706, *P* < 0.05]. Calibration curves of three models in discovery cohort (C) and validation cohort (D). Decision curves analyses of three models in discovery cohort (E) and validation cohort (F). Clinical impact curves of nomogram model in discovery cohort (G) and validation cohort (H).

As revealed by the Graphical Abstract, prospectively, we included 430 participants in discovery cohort (non-MCI = 62, aMCI = 65, AD = 73) and validation cohort (non-MCI = 50, aMCI = 85, AD = 95). Complete demographics, clinical and biochemical data of discovery, and validation cohorts are shown in Supplementary Tables 3, 4. In the discovery cohort, the intersecting features among the Elastic net, RF, XGBoost, and Boruta analyses were considered the most critical features and were visualized by a Venn diagram between AD and non-MCI participants (Supplementary Figure 1). Even though the correlations remained insignificant in the multivariate logistic regression that included Gender and Age as additional confounders, which was considered to eliminate participants’ structure bias caused by Age and Gender between subgroups (Age, Gender, EMPG, Glu, Tau, ALB, HCY, and VB12). The forest plot shows multiple logistic regression results. As demonstrated in Figure 5A, EMPG [odds ratio (OR) = 1.20, 95% confidence interval (CI): 1.07–1.39; *P* = 0.006], Tau (OR = 1.07, 95% CI: 1.04–1.11, *P* < 0.001), and HCY (OR = 1.26, 95% CI: 1.11–1.47, *P* = 0.001) were identified as significantly independent risk factors of AD. ALB level is considered a protective factor (OR = 0.65, 95% CI: 0.45–0.89, *P* < 0.001). Age (*P* = 0.697), Gender (*P* = 0.092), Glu (*P* = 0.209), and VB12 (*P* = 0.125) did not reach statistical significance in multiple logistic regression in the discovery cohort. These conclusions are also available in the validation cohort. In addition, ROC analyses revealed other clinical covariates used to construct the nomogram model, and their performance in differentiating between non-MCI, aMCI, and AD participants was inadequate compared to total Tau (discovery cohort: Supplementary Figure 2; validation cohort: Supplementary Figure 3). The final nomogram model was established using robust variables identified by multiple machine learning algorithms and multivariable logistic regression fitted to the data (Figure 5B). It manifested that Tau level was the dominant factor in the nomogram. The latter was closely followed by EMPG, while other variables had a moderate or low contribution in recognizing AD patients. A corresponding score for each variable at a specific value could be read on the point scale based on a weighting factor and then used to calculate a total score. Overall, the higher the total score of prediction, the more likely it predict AD occurrence. The multipredictor nomogram showed a higher discrimination ability for predicting AD compared with Tau and Clinical model (Age, Gender, EMPG, Glu, ALB, HCY, and VB12) in Figures 6A,B [discovery cohort: AUC, 0.960 (95% CI, 0.928–0.991) vs. 0.799 (95% CI, 0.725–0.873) and 90.8 (95% CI, 0.856–0.960); validation cohort: AUC, 0.938 (95% CI, 0.903–0.973) vs. 0.772 (95% CI, 0.697–0.847), and 91.2 (95% CI, 0.881–0.963), respectively; *P* < 0.001]. Brier Score is another score function that measures the accuracy of probabilistic prediction, and a higher score indicates higher inaccuracy. As for the nomogram, there was good alignment between observed and predicted probability in calibration plots, accompanied by a lower Brier score than total Tau and Clinical model [discovery cohort: 7.3 (95% CI, 4.2–10.5) vs. 17.9 (95% CI, 14.7–21.1) and 11.8 (95% CI, 8.4–15.2); validation cohort: 9.8 (95% CI, 6.9–12.8) vs. 18.1 (95% CI, 15.2–21.1) and 10.9 (95% CI, 7.9–14.0)] (Figures 6C,D). The nomogram had an incremental effect on predictive value than Tau and Clinical model for recognizing AD patients [NRI: 0.486 (95% CI, 0.313–0.659); IDI: 0.415 (95% CI, 0.333–0.496) in the discovery cohort and NRI: 0.294 (95% CI, 0.255–0.362); IDI: 0.378 (95% CI, 0.293–0.463) in the validation cohort, respectively; *P* < 0.001] (Table 1). The decision curves analyses further indicated that the nomogram model was superior to total Tau and clinical model in predicting AD, provided a positive net benefit and was more beneficial than either treat-all or treat-none strategy within the full range of threshold probability (Figures 6E,F). Besides, clinical impact curve analysis was introduced to further evaluate the clinical applicability of this nomogram model (Figures 6G,H). The high-risk population identified by the Nomogram model is consistent with the actual situation, when the threshold probability is greater than 30%. All these results encouraged the nomogram model as a portable tool for AD diagnosis. Given the extremely poor discriminative performance of total Tau in plasma, it is almost impossible to distinguish between aMCI and AD participants. We tried to build another nomogram model to differentiate the AD participants from aMCI. Nomogram were established based on multivariate analysis results in the discovery cohort. Final feature selection was performed by multiple machine learning algorithms (Age, Gender, EMPG, Tau, ALB, HCY, and VB12) (Supplementary Figure 4). Figures 7A,B illustrates the forest plot for multivariable analyses and nomogram for identifying aMCI and AD participants. As demonstrated in Figures 8A,B, the results of discrimination curves (ROC) demonstrated that C-index of the nomogram model was higher than that of total Tau and Clinical model [discovery cohort: 0.813 (95% CI, 0.742–0.885) vs. 0.670 (95% CI, 0.579–0.761) and 0.722 (95% CI, 0.638–0.807); validation cohort: 0.754 (95% CI, 0.683–0.825) vs. 0.652 (95% CI, 0.573–0.732) and 0.706 (95% CI, 0.630–0.782)]. Table 2 shows that the nomogram model had improved NRI and IDI index between AD and aMCI in discovery and validation cohorts. The calibration curves and Brier score revealed good agreement between the estimated outcomes and the actual probability (Figures 8C,D). DCA and clinical impact curves also indicated that the Nomogram model was a favorable clinical tool for differentiating aMCI and AD and outperformed the total Tau and Clinical model within a wide range of threshold probabilities. The proposed model conferred more net benefits compared with both the treat-all-patients scheme and the treat-none scheme in the discovery and validation cohorts (Figures 8E–H).

**TABLE 1:** Evaluating the incremental predictive value and predictive performance of various models with NRI, IDI, and C-index between AD and non-MCI.

	AD vs. non-MCI				
Variable	NRI (95% CI)	*P* value	IDI (95% CI)	*P* value	C-index (95% CI)
**Discovery cohort**					
Tau	Reference		Reference		79.9 (72.5–87.3)
Clinics	0.229 (0.102–0.436)	*P* = 0.003	0.232 (0.115–0.349)	*P* < 0.001	90.8 (85.6–96.0)
Nomogram	0.486 (0.313–0.659)	*P* < 0.001	0.415 (0.333–0.496)	*P* < 0.001	96.0 (92.8–99.1)
**Validation cohort**					
Tau	Reference		Reference		77.2 (69.7–84.7)
Clinics	0.203 (0.096–0.400)	*P* = 0.004	0.326 (0.224–0.428)	*P* < 0.001	91.2 (88.1–96.3)
Nomogram	0.294 (0.255–0.362)	*P* < 0.001	0.378 (0.293–0.463)	*P* < 0.001	93.8 (90.3–97.3)

*NRI, net reclassification index; IDI, integrated discrimination improvement.*

**TABLE 2:** Evaluating the incremental predictive value and predictive performance of various models with NRI, IDI, and C-index between AD and aMCI.

	AD vs. aMCI				
Variable	NRI (95% CI)	*P* value	IDI (95% CI)	*P* value	C-index (95% CI)
**Discovery cohort**					
Tau	Reference		Reference		67.0 (57.9–76.1)
Clinics	0.034 (−0.197 to 0.266)	*P* = 0.071	0.081 (−0.005 to 0.167)	*P* = 0.065	72.2 (63.8–80.7)
Nomogram	0.455 (0.243–0.667)	*P* < 0.001	0.119 (0.105–0.255)	*P* < 0.001	81.3 (74.2–88.5)
Validation cohort					
Tau	Reference		Reference		65.2 (57.3–73.2)
Clinics	0.110 (−0.089 to 0.310)	*P* = 0.280	0.062 (−0.002 to 0.123)	*P* = 0.059	70.6 (63.0–78.2)
Nomogram	0.492 (0.208–0.775)	*P* = 0.001	0.122 (0.074–0.169)	*P* < 0.001	75.4 (68.3–82.5)

*NRI, net reclassification index; IDI, integrated discrimination improvement.*

## Discussion

A research framework for AD has recently been proposed by National Institute on Aging-Alzheimer’s Association (NIA-AA), emphasizing the importance of amyloid-β, Tau, and neuroinflammation in the pathological definition of AD (Jack et al., 2018). Although Aβ- or Tau-based PETs have been developed and promoted, they have not been widely employed in clinical diagnosis, posing a barrier to research framework implementation. Therefore, there is an urgent need to discover convenient biomarkers with early diagnostic significance. Total Tau in CSF has long been acknowledged as a biomarker for AD diagnosis and progression. However, the clinical application of Tau has been hampered by invasiveness and multiple complications of lumbar puncture. Numerous studies have demonstrated that Tau concentration is highly correlated between cerebrospinal fluid and plasma (Jia et al., 2019; Ding et al., 2021). Therefore, accurate quantification of total Tau in plasma would greatly assist in understanding and early diagnosis of this neurodegenerative disease. However, Tau concentration in plasma is usually below the limits of conventional ELISA performance (Jongbloed et al., 2013). Increased research has developed ultra-micro quantitative analysis based on some novel technologies such as single-molecule array (Simoa), immunomagnetic reduction (IMR), etc. (Rissin et al., 2010; Rubenstein et al., 2014). Promising results exist for plasma total Tau (T-tau) and phosphorylated Tau (P-tau) measured using a sensitive immunoassay with high-precision detection (Ding et al., 2021; Jiao et al., 2021; Jordan et al., 2021). While these preliminary results are encouraging, their dissemination is constrained by the need for specialized testing equipment and complicated operating procedures.

This study developed a novel approach based on immunochemistry (CLIA) to quantify total Tau in human plasma. CLIA immunoassay was conducted and relied on specific antibodies, magnetic nanoparticles with high antibody loading capacity, BAS cascading amplification systems, and acridyl esters with excellent luminescence efficiency. Under optimal experimental conditions, the quantitative analysis demonstrated satisfying analytical performance, including considerable sensitivity (5.16 pg/mL) and an appropriate linear range (7.80–250 pg/mL). The results demonstrated that our proposed CLIA could be applied for sensitive quantification of total Tau in actual plasma samples. Multiple ROC curves indicated that plasma Tau could more accurately identify participants in different groups (aMCI vs. non-MCI: 0.6909; AD vs. non-MCI: 0.7993; AD vs. aMCI: 0.6698). Therefore, it is expected to be considered a fast, simple, and convenient tool that can provide highly accurate results and achieve sensitive concentration analysis for total Tau in plasma. What needs to be acknowledged is that single factors often face significant challenges in predicting specific clinical outcomes.

The development of blood-based biomarkers for Alzheimer’s disease pathology as tools for screening the general population is essential but persists controversies. Some researchers highlight that total Tau in plasma demonstrated similar predictive validity compared with total Tau in CSF for determining risk for incipient dementia (Pase et al., 2019). Another study found that higher levels of plasma total tau were associated with an increased risk of MCI and with global- and domain-specific cognitive decline on cognitive testing in a manner independent from amyloid PET imaging (Mielke et al., 2017). A total of 42 studies on tau proteins in blood were eligible in a systematic review and meta-analysis, which revealed that different measure methods (ELISA, IMR, and SIMOA) showed equally diagnostic values from controls to aMCI patients to AD patients (Qu et al., 2021). However, some researchers hold different opinions. For example, T-tau has not discriminated between healthy controls and MCI in multiple studies, where elevated levels were only observed in AD dementia, with considerable overlap between groups (Zetterberg et al., 2013; Mattsson et al., 2016; Mielke et al., 2018). The predictive utility also appears limited, as plasma t-tau has not significantly predicted conversion from MCI to dementia (Zetterberg et al., 2013; Mielke et al., 2017). Given the ambiguous findings for plasma measurements of T-tau, further investigation is crucial to validate these measurements as *in vivo* biomarkers for AD. Overall, our study adds to the current literature in suggesting that plasma tau could represent a valuable biomarker to include in panels for AD clinical studies in addition to blood biomarkers. More research is needed to better understand the molecular pathways responsible for the accumulation, clearance, and catabolism of Tau in plasma, the relationship of Tau between plasma and CSF, and their association with cognitive dysfunction and blood-brain barrier permeability measures.

In this study, EMPG and HCY have also been considered as risk factors for AD patients through multivariate logistic regression, consistent with previous studies (Lin et al., 2014; Chen et al., 2020; Lu et al., 2020). However, as diagnostic biomarkers for AD, they have not fully satisfied the clinical prognostic needs. Given the complexity of pathological progression, it also seems unlikely to have an ideal diagnostic biomarker that can independently diagnose AD. However, since these biomarkers represent different aspects of AD, a combination of them might provide higher performance than each of them alone. To solve these problems, we have successfully constructed and verified a combined model including Tau and clinical variables, imparting a better performance in diagnosing AD patients. In this study, we found that a well-fitted multivariate model could independently recognize AD from aMCI participants, and combined predictors enabled the selection of patients with a higher risk of Alzheimer’s disease. Besides, we have developed a visual nomogram that can easily use this predictive model in clinical practice. The proposed nomogram exhibited better performance than those based on only a single predictor variable. Specifically, the nomogram model has a C-index of 0.960 in distinguishing AD patients and elderly non-MCI people, and a C-index of 0.813 in recognizing the AD from aMCI participants.

Although the results were promising, some limitations remained in the present study. Firstly, the ultrasensitive assay of total Tau in plasma has not been extensively tested, leading to high heterogeneity across different centers, resulting in these divergent results. Secondly, all patients are from the same medical center, leading to a potentially overly optimistic model. More convincing data from multiple medical centers should allow further evaluation of diagnostic models before generalizing the results to other medical centers. Finally, long-term conversion of aMCI cannot yet be clearly defined due to the lack of continuous follow-up of radiological images or neurophysiology assessment. Moreover, it is widely recognized that treatment decisions would greatly facilitate intervention decisions, delaying aMCI conversion or AD progression.

## Conclusion

In conclusion, the presented results validated the possibility of total Tau in plasma as a candidate for AD diagnosis based on an optimized CLIA procedure. We also provide a well-fitted multipredictor nomogram to assist clinical diagnosis and prediction.

## Data Availability Statement

The original contributions presented in the study are included in the article/Supplementary Material, further inquiries can be directed to the corresponding authors.

## Ethics Statement

The study was approved by the Human Research Ethics Committee of The Second Affiliated Hospital of Zhejiang University School of Medicine. All participants and/or legal guardians have been fully informed and completed a written consent form.

## Author Contributions

LZ and ZT conceived and designed the study. LZ and WL analyzed the datasets and were responsible for the writing of this manuscript and under the responsibility of the production of figures and tables. DW, YC, XW, and YD searched a large number of literature and were responsible for reference compilation. All authors reviewed and considered the final manuscript.

## Conflict of Interest

The authors declare that the research was conducted in the absence of any commercial or financial relationships that could be construed as a potential conflict of interest.

## Publisher’s Note

All claims expressed in this article are solely those of the authors and do not necessarily represent those of their affiliated organizations, or those of the publisher, the editors and the reviewers. Any product that may be evaluated in this article, or claim that may be made by its manufacturer, is not guaranteed or endorsed by the publisher.

## Footnotes

1
https://github.com/harrelfe/rms

2
http://www.r-project.org/

## Abbreviations

AD: Alzheimer’s disease
aMCI: amnestic mild cognitive Impairment
MMSE: mini-mental state examination
CSF: cerebrospinal fluid
PET: positron emission tomography
HCY: homocysteine
EMPG: estimated mean plasma glucose concentration
Glu: random fasting blood glucose
ALB: albumin
VB12: vitamin B12
AFU: α -L-fucosidase
ALP: alkaline phosphatase
ALT: alanine aminotransferase
AST: aspartate transaminase
BUN: blood urea nitrogen
CHE: cholinesterase
CRP: C-reactive protein
Cr: endogenous creatinine clearance rate
CYSC: cystatin C
DB: direct bilirubin
FER: ferritin
FFA: free fatty acid
FH: folate
FT3: free Triiodothyronine
FT4: free thyroxine
GGT: gamma glutamyl transferase
GLOB: globulin
HBA1: hemoglobin A1
HBA1C: hemoglobin A1c
HBF: hemoglobin F
HCT: red blood cell specific volume
HDL: high density lipoprotein
HGB: hemoglobin
IB: indirect bilirubin
LDL: low-density lipoprotein
PLT: platelet count
TB: total bilirubin
TC: serum total cholesterol
TG: triglyceride
TT3: total triiodothyronine
TT4: thyroxine
UA: uric acid
TSH: thyroid stimulating hormone.
