# Supplementary material for: Machine Learning Reveals a Multipredictor Nomogram for Diagnosing the Alzheimer’s Disease Based on Chemiluminescence Immunoassay for Total Tau in Plasma
Source: Front Aging Neurosci. 2022 May 13;14:863673. doi: 10.3389/fnagi.2022.863673 (PMC9136081; doi:10.3389/fnagi.2022.863673)
Supplement: Supplementary file 1 [file Data_Sheet_1.pdf]

Supplementary Material

Machine learning reveals a multipredictor nomogram for diagnosing the Alzheimer's disease based on chemiluminescence immunoassay for total Tau in plasma

TABLE S1. Precision verification for CLIA

Standard concentration (pg/mL)	Average detected concentration (pg/mL)	Recovery Range (%)	Inter-day RSD (%)	Intra-day RSD (%)	Total RSD (%)
10	10.38±1.27	85%-111%	9.54%	9.12%	9.68%
50	50.32±1.81	94%-104%	3.88%	3.63%	4.02%
100	100.23±2.24	98%-103%	1.91%	2.21%	2.88%

Abbreviation: RSD, relative standard deviation.

TABLE S2. Comparison of the detection performance of other analytical techniques with CLIA

Detection Technologies	LOD	Linear Range	CV (%)	Contributors
ELISA	16 pg/mL	40.6-2600 pg/mL	2.7-5.5%	Abcam Tau ELISA kit
Simoa	0.019 pg/mL	0.06-360 pg/mL	4.1-7.5%	Matthew P.P, et al(Pase et al.,2019)
Chip-based optical	15.6 pg/mL	62.5-500 pg/mL	4.8-6.3%	Song C, et al(Song et al.,2018)
SERS	0.15 pg/mL	0-6 pg/mL	3.2-5.0%	Demeritte T, et al(Demeritte et al.,2015)
Lumipulse G	2.75 pg/mL	11-1496 pg/mL	0.9-3.5%	Bayart J.L, et al(Bayart et al.,2019)
xMAP	6.2 pg/mL	25-1500 pg/mL	2.9-4.8%	Kang J.H, et al(Kang et al.,2012)
CLIA	5.16 pg/mL	7.80-250 pg/mL	2.9-9.7%	Our work

Abbreviation: ELISA, enzyme linked immunosorbent assay; SERS, Surface-Enhanced Raman Scattering; xMAP, flexible Multi-Analyte Profiling.

TABLE S3. Baseline Characteristics of the participants in the discovery cohort.

Variables	Control (N=62)	aMCI (N=65)	Alzheimer (N=73)	P-value
**Gender**				
Female	33 (53.2%)	41 (63.1%)	47 (64.4%)	0.365
Male	29 (46.8%)	24 (36.9%)	26 (35.6%)	
**Age (years)**				
Mean (SD)	67.2 (8.44)	67.5 (9.96)	73.1 (9.92)	0.344
Median [Min, Max]	65.5 [51.0, 88.0]	68.0 [48.0, 91.0]	74.0 [48.0, 90.0]	
**Tau (pg/mL)**				
Mean (SD)	20.8 (16.8)	34.4 (23.8)	57.2 (40.1)	<0.001
Median [Min, Max]	15.9 [4.87, 73.7]	28.3 [5.16, 102]	48.6 [5.16, 169]	
**AFU (U/L)**				
Mean (SD)	24.3 (3.79)	25.4 (4.52)	25.5 (5.26)	0.0326
Median [Min, Max]	24.5 [15.0, 32.0]	25.0 [12.0, 38.0]	25.0 [18.0, 42.0]	
**ALB (g/L)**				
Mean (SD)	42.7 (2.08)	41.0 (2.45)	40.8 (1.86)	0.0744

Median [Min, Max]	42.9 [37.2, 46.6]	41.2 [36.2, 48.0]	41.0 [36.8, 46.2]	
**ALP (U/L)**				
Mean (SD)	79.6 (17.8)	82.9 (19.7)	80.2 (20.5)	0.534
Median [Min, Max]	79.0 [47.0, 119]	83.5 [48.0, 134]	76.0 [31.0, 134]	
**ALT (U/L)**				
Mean (SD)	19.0 (6.48)	17.3 (7.07)	18.4 (10.0)	<0.001
Median [Min, Max]	18.0 [9.00, 44.0]	16.7 [6.00, 45.0]	16.0 [10.0, 66.0]	
**AST (U/L)**				
Mean (SD)	23.6 (5.40)	22.7 (6.09)	24.7 (10.0)	<0.001
Median [Min, Max]	23.5 [12.0, 38.0]	21.8 [11.0, 49.0]	22.0 [15.0, 85.0]	
**BUN (mmol/L)**				
Mean (SD)	5.02 (0.968)	5.54 (1.42)	5.67 (2.45)	<0.001
Median [Min, Max]	4.99 [3.29, 7.68]	5.44 [3.15, 9.89]	5.02 [2.03, 19.2]	
**CHE (U/L)**				
Mean (SD)	4.89 (0.672)	5.16 (0.929)	5.24 (0.887)	0.0294
Median [Min, Max]	4.70 [3.78, 6.87]	4.98 [3.21, 7.94]	5.12 [3.31, 7.28]	
**CRP (mg/L)**				
Mean (SD)	3.72 (0.776)	3.51 (2.03)	3.75 (2.38)	<0.001
Median [Min, Max]	3.78 [0.600, 6.60]	3.65 [0.200, 11.2]	3.69 [0.300, 13.8]	
**Cr (μmol/L)**				
Mean (SD)	66.5 (12.1)	71.8 (15.6)	73.5 (23.1)	<0.001
Median [Min, Max]	66.0 [44.0, 101]	68.0 [46.0, 116]	68.0 [42.0, 190]	
**CYSC**				
Mean (SD)	1.08 (0.178)	1.19 (0.286)	1.17 (0.314)	<0.001
Median [Min, Max]	1.06 [0.780, 1.52]	1.15 [0.740, 2.14]	1.10 [0.710, 2.22]	
**DB (μmol/L)**				
Mean (SD)	2.40 (0.816)	2.47 (0.830)	2.77 (1.05)	0.0609
Median [Min, Max]	2.30 [1.20, 5.60]	2.30 [1.00, 6.50]	2.70 [1.00, 7.60]	
**EMPG (mmol/L)**				
Mean (SD)	6.07 (0.486)	6.50 (0.693)	6.85 (0.819)	<0.001
Median [Min, Max]	6.10 [5.10, 7.10]	6.50 [5.20, 8.70]	6.80 [5.50, 9.20]	
**FER (μg/L)**				
Mean (SD)	123 (71.1)	197 (177)	168 (162)	<0.001
Median [Min, Max]	112 [30.2, 611]	144 [21.5, 1110]	122 [10.5, 1200]	
**FFA (μmol/L)**				
Mean (SD)	170 (95.9)	224 (158)	301 (260)	<0.001
Median [Min, Max]	144 [112, 770]	167 [50.0, 833]	174 [13.6, 929]	
**FH (nmol/L)**				
Mean (SD)	23.9 (9.24)	26.6 (12.7)	26.0 (11.3)	0.0472
Median [Min, Max]	21.0 [6.91, 54.5]	23.8 [5.71, 54.5]	23.0 [7.30, 54.5]	
**FT3 (pmol/L)**				
Mean (SD)	4.96 (0.539)	4.46 (0.510)	4.51 (0.529)	0.908
Median [Min, Max]	4.92 [3.29, 6.02]	4.38 [3.47, 5.50]	4.49 [3.10, 6.30]	

**FT4 (pmol/L)**				
Mean (SD)	13.3 (1.52)	12.7 (1.36)	12.6 (2.26)	<0.001
Median [Min, Max]	13.2 [10.7, 17.5]	12.6 [10.4, 18.7]	12.7 [7.01, 18.2]	
**GGT (U/L)**				
Mean (SD)	22.3 (9.46)	21.0 (11.2)	24.0 (16.4)	<0.001
Median [Min, Max]	21.0 [9.00, 55.0]	19.0 [10.0, 98.0]	20.0 [10.0, 110]	
**Glu (mmol/L)**				
Mean (SD)	5.38 (0.530)	5.79 (0.776)	6.24 (1.17)	<0.001
Median [Min, Max]	5.34 [4.17, 6.80]	5.65 [4.29, 8.81]	6.07 [4.21, 10.1]	
**GLOB (g/L)**				
Mean (SD)	28.5 (3.62)	28.9 (4.69)	28.5 (3.72)	0.0645
Median [Min, Max]	28.4 [19.4, 36.3]	28.1 [21.5, 44.2]	28.6 [20.4, 38.2]	
**HBA1 (%)**				
Mean (SD)	6.72 (0.373)	6.87 (0.506)	6.98 (0.603)	<0.001
Median [Min, Max]	6.70 [6.10, 7.90]	6.80 [6.18, 8.40]	7.00 [5.72, 8.80]	
**HBA1c (%)**				
Mean (SD)	5.63 (0.359)	5.72 (0.418)	5.86 (0.586)	<0.001
Median [Min, Max]	5.60 [5.00, 7.00]	5.60 [5.10, 6.80]	5.80 [4.64, 7.60]	
**HBF (%)**				
Mean (SD)	0.574 (0.208)	0.718 (0.456)	0.577 (0.282)	<0.001
Median [Min, Max]	0.600 [0.200, 1.30]	0.600 [0.200, 2.38]	0.500 [0.200, 1.50]	
**HCT (%100)**				
Mean (SD)	0.442 (0.0320)	0.405 (0.0337)	0.393 (0.0396)	0.183
Median [Min, Max]	0.440 [0.380, 0.500]	0.403 [0.300, 0.506]	0.390 [0.290, 0.530]	
**HDL (mmol/L)**				
Mean (SD)	1.52 (0.327)	1.37 (0.358)	1.42 (0.507)	<0.001
Median [Min, Max]	1.49 [1.08, 2.89]	1.32 [0.620, 2.95]	1.28 [0.620, 3.49]	
**HGB (g/L)**				
Mean (SD)	145 (12.1)	135 (11.2)	129 (13.0)	0.457
Median [Min, Max]	144 [116, 167]	134 [116, 173]	128 [92.0, 178]	
**IB**				
Mean (SD)	10.7 (3.41)	10.0 (4.54)	11.2 (4.66)	0.0321
Median [Min, Max]	10.4 [5.30, 20.8]	9.10 [4.50, 36.8]	11.1 [1.25, 28.5]	
**LDL (mmol/L)**				
Mean (SD)	2.43 (0.780)	2.46 (0.700)	2.49 (0.700)	0.599
Median [Min, Max]	2.49 [0.800, 4.33]	2.42 [1.12, 4.29]	2.33 [0.940, 4.21]	
**PLT (*10^9/L)**				
Mean (SD)	209 (43.5)	208 (40.8)	200 (52.6)	0.0895
Median [Min, Max]	204 [105, 313]	203 [121, 309]	200 [102, 320]	
**TB (µmol/L)**				
Mean (SD)	13.0 (3.91)	12.6 (5.42)	14.0 (5.66)	0.00928
Median [Min, Max]	12.6 [6.50, 24.3]	11.5 [6.10, 43.3]	13.6 [6.20, 36.1]	
**TC (mmol/L)**				
Mean (SD)	4.58 (1.12)	4.54 (0.914)	4.88 (0.872)	0.0899

Median [Min, Max]	4.61 [2.05, 6.42]	4.63 [2.63, 6.70]	4.90 [2.74, 6.85]	
**TP (g/L)**				
Mean (SD)	72.5 (5.48)	66.2 (8.30)	65.2 (7.46)	0.00492
Median [Min, Max]	72.6 [46.3, 82.0]	68.3 [40.6, 82.0]	66.7 [47.5, 77.8]	
**TG (mmol/L)**				
Mean (SD)	1.26 (0.588)	1.85 (0.805)	1.69 (0.721)	0.0496
Median [Min, Max]	1.14 [0.310, 2.99]	1.73 [0.670, 4.60]	1.64 [0.460, 4.65]	
**TT3 (nmol/L)**				
Mean (SD)	1.74 (0.294)	1.53 (0.254)	1.55 (0.314)	0.223
Median [Min, Max]	1.76 [1.09, 2.47]	1.51 [1.05, 2.26]	1.49 [0.940, 2.25]	
**TT4 (nmol/L)**				
Mean (SD)	106 (22.9)	99.2 (19.5)	95.8 (23.0)	0.324
Median [Min, Max]	108 [57.9, 152]	99.6 [51.7, 173]	98.2 [51.5, 144]	
**UA (μmol/L)**				
Mean (SD)	296 (60.6)	301 (76.6)	311 (95.9)	0.00124
Median [Min, Max]	293 [189, 427]	297 [180, 562]	303 [157, 537]	
**VB12 (pmol/L)**				
Mean (SD)	433 (209)	366 (177)	306 (151)	0.0282
Median [Min, Max]	429 [135, 1040]	328 [114, 881]	261 [106, 740]	
**TSH (U/L)**				
Mean (SD)	1.78 (0.902)	1.74 (1.46)	1.58 (0.919)	<0.001
Median [Min, Max]	1.74 [0.0500, 4.11]	1.33 [0.240, 9.08]	1.38 [0.180, 5.20]	
**HCY (μmol/L)**				
Mean (SD)	13.5 (3.62)	15.4 (5.08)	17.1 (6.28)	<0.001
Median [Min, Max]	12.4 [8.60, 25.2]	14.3 [9.40, 34.2]	14.7 [8.40, 34.9]	

TABLE S4. Baseline Characteristics of the participants in the validation cohort.

Variables	Control (N=50)	aMCI (N=85)	Alzheimer (N=95)	P-value
**Gender**				
Female	15 (30.0%)	43 (50.6%)	61 (64.2%)	<0.001
Male	35 (70.0%)	42 (49.4%)	34 (35.8%)	
**Age (years)**				
Mean (SD)	66.2 (7.40)	68.8 (8.96)	71.7 (9.72)	0.108
Median [Min, Max]	64.5 [57.0, 84.0]	68.0 [45.0, 88.0]	72.0 [49.0, 92.0]	
**Tau (pg/mL)**				
Mean (SD)	31.5 (17.5)	43.2 (25.6)	59.3 (31.3)	<0.001
Median [Min, Max]	29.3 [5.16, 72.6]	38.4 [5.16, 111]	54.5 [9.59, 137]	
**AFU (U/L)**				
Mean (SD)	27.8 (6.18)	25.8 (5.16)	26.0 (4.99)	0.192
Median [Min, Max]	27.0 [17.0, 50.0]	25.0 [17.0, 40.0]	26.0 [17.0, 38.0]	
**ALB (g/L)**				
Mean (SD)	41.6 (2.24)	40.7 (2.06)	40.0 (2.18)	0.78

Median [Min, Max]	41.9 [36.1, 46.1]	41.2 [34.8, 44.9]	40.2 [32.7, 44.4]	
**ALP (U/L)**				
Mean (SD)	80.0 (18.5)	81.5 (23.0)	81.8 (27.7)	0.00687
Median [Min, Max]	76.5 [40.0, 128]	76.0 [45.0, 171]	81.0 [27.0, 206]	
**ALT (U/L)**				
Mean (SD)	18.2 (6.99)	16.0 (7.36)	17.1 (8.84)	0.0963
Median [Min, Max]	17.0 [9.00, 39.0]	14.0 [6.00, 56.0]	15.0 [5.00, 61.0]	
**AST (U/L)**				
Mean (SD)	22.2 (5.31)	20.8 (4.95)	23.2 (7.29)	<0.001
Median [Min, Max]	21.0 [12.0, 35.0]	20.0 [12.0, 43.0]	23.0 [11.0, 48.0]	
**BUN (mmol/L)**				
Mean (SD)	5.32 (1.08)	5.48 (1.19)	5.66 (1.50)	0.0132
Median [Min, Max]	5.05 [2.97, 7.36]	5.32 [2.35, 8.87]	5.44 [3.09, 9.85]	
**CHE (U/L)**				
Mean (SD)	5.13 (1.00)	5.97 (1.49)	5.77 (1.58)	0.00247
Median [Min, Max]	5.18 [2.45, 7.51]	5.60 [3.22, 9.71]	5.73 [1.79, 9.71]	
**CRP (mg/L)**				
Mean (SD)	3.91 (1.47)	2.98 (2.42)	2.76 (2.12)	0.00115
Median [Min, Max]	4.04 [0.300, 8.50]	2.70 [0.200, 14.3]	2.70 [0.200, 9.90]	
**Cr (μmol/L)**				
Mean (SD)	70.3 (13.4)	69.9 (15.0)	69.9 (18.5)	0.0224
Median [Min, Max]	70.9 [45.0, 106]	69.4 [38.9, 136]	67.0 [38.9, 136]	
**CYSC**				
Mean (SD)	1.13 (0.396)	1.10 (0.309)	1.09 (0.334)	0.139
Median [Min, Max]	1.07 [0.600, 3.19]	1.06 [0.600, 2.31]	1.00 [0.400, 2.49]	
**DB (μmol/L)**				
Mean (SD)	2.67 (1.04)	2.72 (1.81)	2.96 (1.91)	<0.001
Median [Min, Max]	2.50 [1.40, 7.90]	2.30 [0.991, 15.4]	2.60 [1.00, 15.4]	
**EMPG (mmol/L)**				
Mean (SD)	6.58 (0.643)	7.02 (1.11)	7.54 (1.33)	<0.001
Median [Min, Max]	6.50 [5.80, 9.50]	6.80 [5.20, 11.2]	7.10 [5.50, 12.0]	
**FER (μg/L)**				
Mean (SD)	170 (71.1)	189 (130)	171 (116)	<0.001
Median [Min, Max]	154 [40.9, 383]	143 [16.1, 737]	145 [10.4, 652]	
**FFA (μmol/L)**				
Mean (SD)	258 (168)	342 (257)	380 (287)	<0.001
Median [Min, Max]	203 [28.0, 753]	251 [20.0, 1630]	321 [20.0, 1630]	
**FH (nmol/L)**				
Mean (SD)	24.4 (6.39)	27.2 (11.2)	27.2 (11.9)	<0.001
Median [Min, Max]	22.8 [9.87, 44.7]	24.1 [6.68, 67.8]	23.4 [9.87, 58.2]	
**FT3 (pmol/L)**				
Mean (SD)	4.57 (0.518)	4.19 (0.668)	4.11 (0.563)	0.0986
Median [Min, Max]	4.59 [3.37, 5.70]	4.18 [2.90, 7.10]	4.05 [2.68, 5.79]	
**FT4 (pmol/L)**				

Mean (SD)	12.2 (2.12)	12.8 (1.49)	12.4 (1.93)	0.0119
Median [Min, Max]	12.2 [7.43, 16.6]	12.6 [8.70, 18.3]	12.7 [6.26, 15.9]	
**GGT (U/L)**				
Mean (SD)	26.3 (15.4)	24.6 (18.2)	25.0 (19.1)	0.239
Median [Min, Max]	20.0 [11.0, 77.0]	19.0 [9.00, 104]	19.0 [9.00, 141]	
**Glu (mmol/L)**				
Mean (SD)	5.56 (0.799)	5.86 (0.922)	6.16 (1.04)	0.113
Median [Min, Max]	5.38 [4.13, 9.16]	5.67 [4.54, 9.31]	6.10 [4.54, 10.1]	
**GLOB (g/L)**				
Mean (SD)	28.5 (3.69)	27.5 (3.53)	27.9 (4.27)	0.181
Median [Min, Max]	28.8 [19.1, 39.9]	27.7 [19.1, 36.2]	27.7 [19.6, 40.9]	
**HBA1 (%)**				
Mean (SD)	7.14 (0.441)	7.11 (1.31)	7.32 (1.17)	<0.001
Median [Min, Max]	7.10 [6.40, 8.70]	7.00 [4.54, 15.5]	7.10 [5.04, 10.7]	
**HBA1c (%)**				
Mean (SD)	5.93 (0.420)	6.03 (1.16)	6.15 (0.891)	<0.001
Median [Min, Max]	5.80 [5.30, 7.60]	5.80 [4.40, 14.1]	5.90 [4.50, 9.20]	
**HBF (%)**				
Mean (SD)	0.706 (0.311)	0.694 (0.356)	0.687 (0.305)	0.31
Median [Min, Max]	0.700 [0.300, 2.20]	0.600 [0.300, 2.80]	0.600 [0.200, 2.20]	
**HCT (%100)**				
Mean (SD)	0.432 (0.0362)	0.408 (0.0391)	0.401 (0.0380)	0.84
Median [Min, Max]	0.441 [0.355, 0.501]	0.409 [0.294, 0.492]	0.404 [0.216, 0.485]	
**HDL (mmol/L)**				
Mean (SD)	1.43 (0.385)	1.42 (0.313)	1.51 (0.439)	0.00765
Median [Min, Max]	1.38 [0.810, 2.42]	1.39 [0.690, 2.49]	1.37 [0.710, 2.73]	
**HGB (g/L)**				
Mean (SD)	143 (13.5)	135 (14.7)	131 (13.6)	0.698
Median [Min, Max]	145 [115, 171]	135 [95.0, 169]	131 [73.0, 160]	
**IB**				
Mean (SD)	12.3 (3.48)	10.9 (4.07)	11.9 (4.96)	0.0148
Median [Min, Max]	11.8 [5.90, 19.4]	10.2 [6.10, 25.5]	10.6 [5.00, 34.6]	
**LDL (mmol/L)**				
Mean (SD)	2.78 (0.756)	2.44 (0.881)	2.58 (0.840)	0.495
Median [Min, Max]	2.81 [0.940, 4.36]	2.19 [0.940, 4.84]	2.43 [1.04, 4.36]	
**PLT (*10^9/L)**				
Mean (SD)	202 (61.9)	208 (52.8)	199 (48.2)	0.125
Median [Min, Max]	186 [112, 385]	204 [112, 341]	194 [104, 334]	
**TB (μmol/L)**				
Mean (SD)	15.4 (5.33)	14.0 (6.08)	14.8 (5.96)	0.58
Median [Min, Max]	14.6 [7.30, 39.2]	12.5 [7.80, 42.0]	14.3 [6.10, 40.9]	
**TC (mmol/L)**				
Mean (SD)	5.12 (0.910)	4.77 (1.14)	4.84 (1.17)	0.134

Median [Min, Max]	5.13 [3.03, 6.88]	4.69 [2.17, 7.80]	4.86 [2.27, 7.56]	
**TP (g/L)**				
Mean (SD)	70.1 (4.09)	69.0 (3.56)	68.4 (4.26)	0.231
Median [Min, Max]	70.2 [57.3, 82.1]	69.4 [56.8, 77.5]	68.8 [56.6, 81.4]	
**TG (mmol/L)**				
Mean (SD)	1.30 (0.576)	1.34 (0.571)	1.29 (0.630)	0.6
Median [Min, Max]	1.16 [0.440, 3.02]	1.27 [0.250, 4.05]	1.16 [0.150, 3.00]	
**TT3 (nmol/L)**				
Mean (SD)	1.59 (0.279)	1.40 (0.230)	1.44 (0.281)	0.132
Median [Min, Max]	1.59 [1.01, 2.31]	1.40 [0.890, 1.94]	1.42 [0.990, 2.43]	
**TT4 (nmol/L)**				
Mean (SD)	95.0 (21.9)	97.5 (17.4)	93.0 (18.8)	0.187
Median [Min, Max]	95.2 [50.5, 133]	95.7 [67.0, 162]	94.0 [46.5, 137]	
**UA (μmol/L)**				
Mean (SD)	365 (99.4)	330 (88.5)	326 (112)	0.0896
Median [Min, Max]	339 [202, 649]	318 [162, 569]	304 [154, 649]	
**VB12 (pmol/L)**				
Mean (SD)	530 (251)	429 (251)	363 (141)	<0.001
Median [Min, Max]	487 [160, 1210]	356 [142, 1480]	326 [150, 898]	
**TSH (U/L)**				
Mean (SD)	1.84 (1.15)	1.53 (0.851)	1.62 (1.05)	0.0398
Median [Min, Max]	1.65 [0.520, 7.70]	1.33 [0.260, 5.83]	1.42 [0.0900, 6.32]	
**HCY (μmol/L)**				
Mean (SD)	12.3 (1.99)	14.6 (4.36)	16.0 (4.75)	<0.001
Median [Min, Max]	12.2 [9.30, 21.4]	13.6 [9.30, 37.3]	14.8 [9.30, 33.3]	

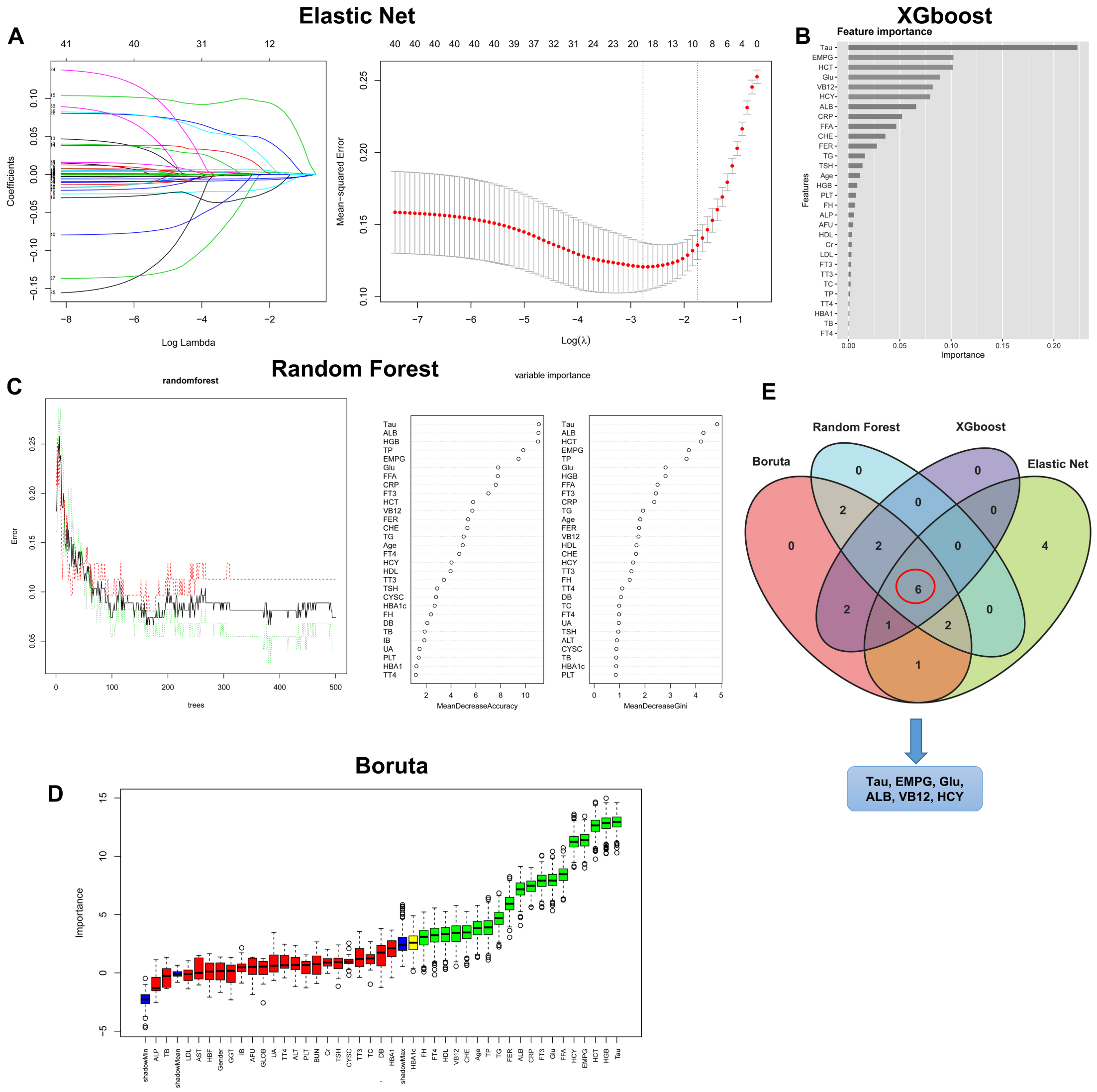

Supplementary Figure S1. Machine learning algorithms for identifying the most robust subgroup relevant features. (**A**) Elastic Net analysis. Left panel: Coefficients of the total 42 features. Right panel: Partial likelihood deviance as a function of regularization parameter λ for 10-fold-cross validation in the discovery cohort. (**B**) The importance of relevant variables screened by XGboost algorithm. (**C**) Random forest algorithm. Left panel: Associations between tree numbers and the error rate between AD and non-MCI and out-of-bag (OOB) samples. Right panel: The most important features identified by random forest. (**D**) The important variables identified by Boruta algorithm. (**E**) Venn diagram identified seven most robust subgroup-specific features that were shared by four machine learning algorithms.

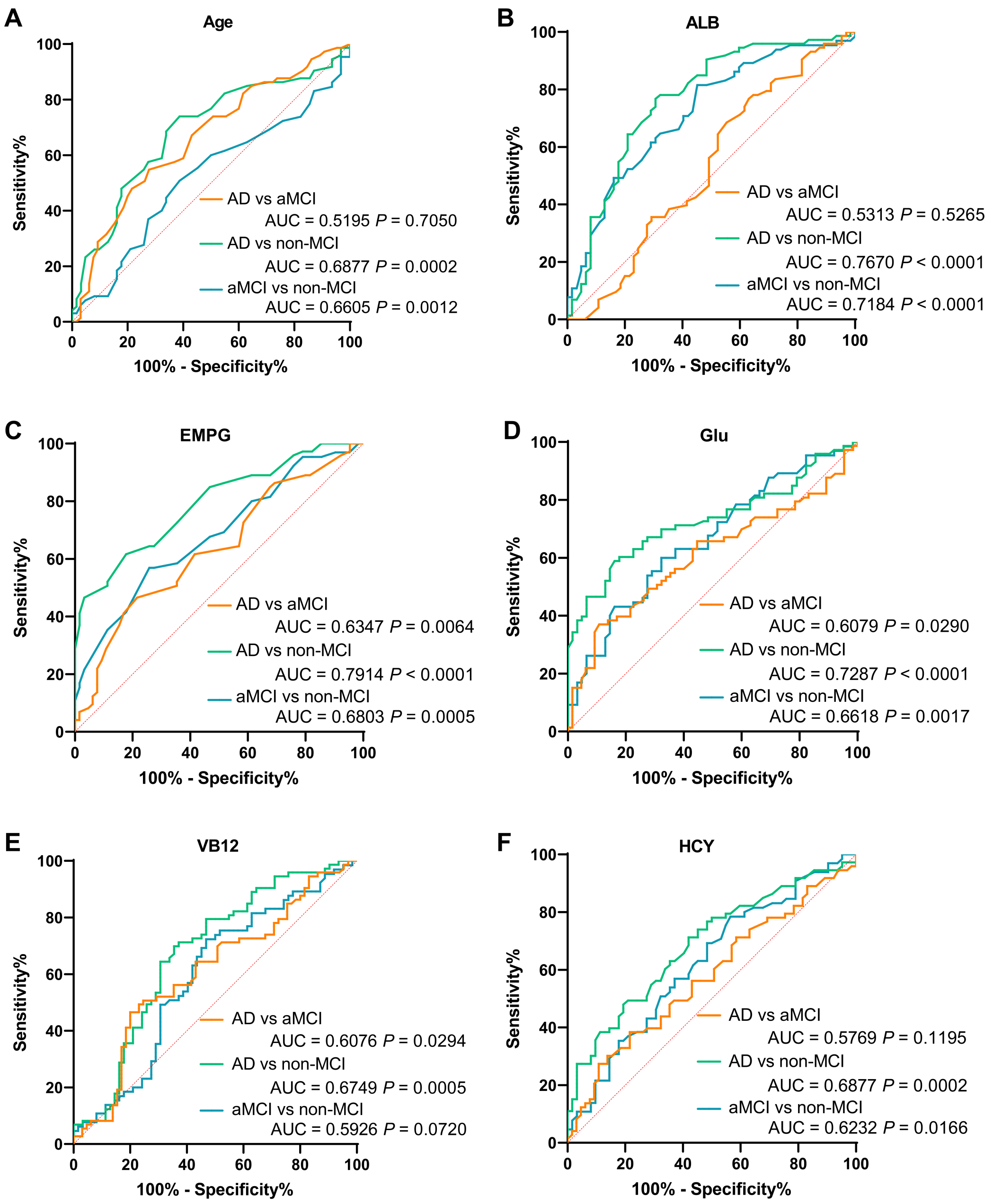

Supplementary Figure S2. Performance of other clinical variables in the discovery cohort. **A-F** shows ROC analyses of Age (**A**), ALB (**B**), EMPG (**C**), Glu (**D**), VB12 (**E**), HCY (**F**) among AD patients, aMCI patients, and non-MCI participants.

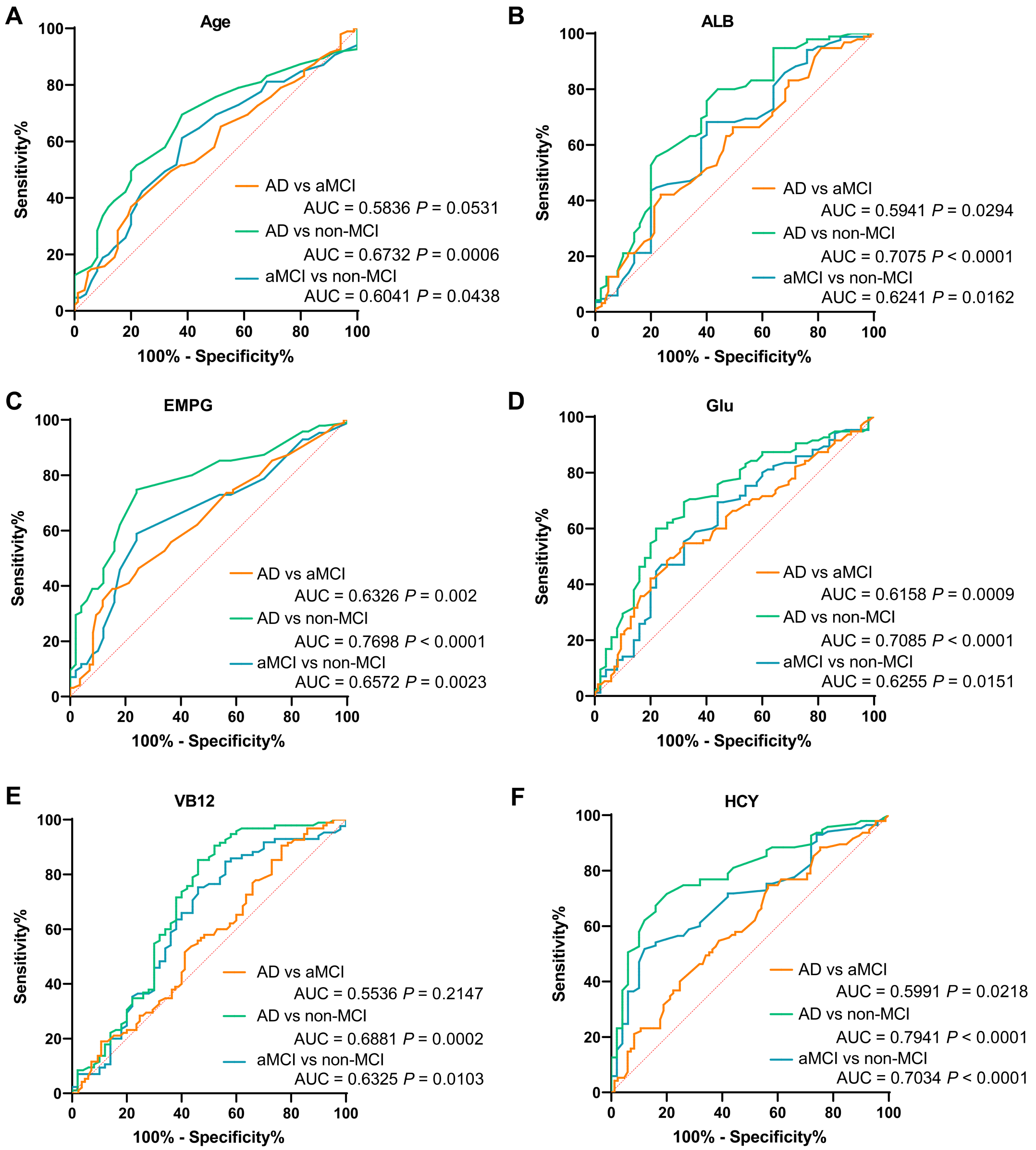

Supplementary Figure S3. Performance of other clinical variables in the validation cohort. **A-F** shows ROC analyses of Age (**A**), ALB (**B**), EMPG (**C**), Glu (**D**), VB12 (**E**), HCY (**F**) among AD patients, aMCI patients, and non-MCI participants.

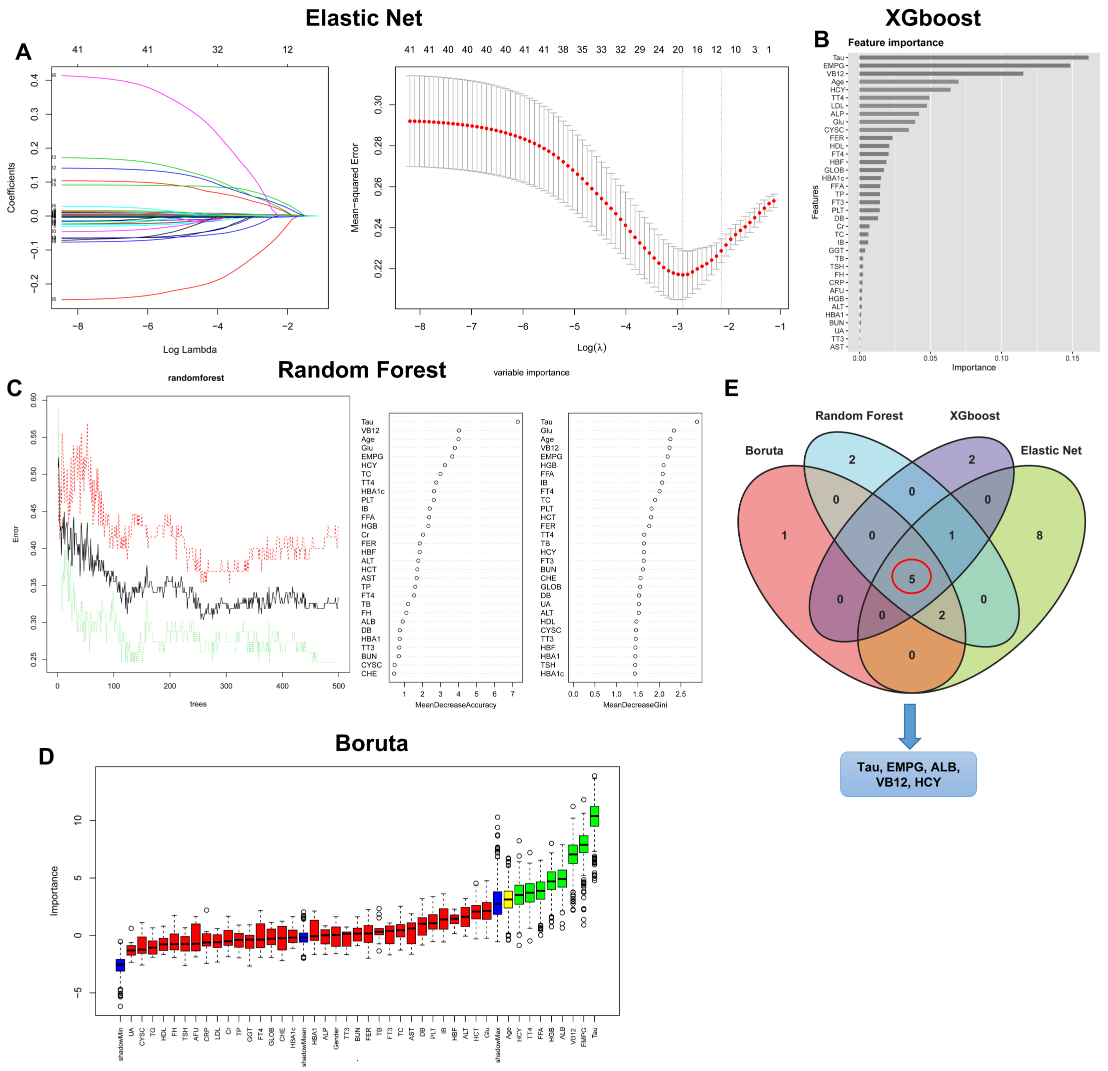

Supplementary Figure S4. Machine learning algorithms for identifying the most robust features between AD and aMCI participants. (**A**) Elastic Net analysis. (**B**) The importance of relevant variables screened by XGboost algorithm. (**C**) Random forest algorithm. (**D**) The important variables identified by Boruta algorithm. (**E**) Venn diagram identified seven most robust subgroup-specific features that were shared by four machine learning algorithms.
